# Efficacy and safety of modified medium-chain triglyceride ketogenic diet in patients with drug-resistant epilepsy

**DOI:** 10.1186/s42494-024-00150-x

**Published:** 2024-03-06

**Authors:** Hua Li, Yao Wang, Jing Guo, Peiqi Zhang, Zheng Xu, Kai Peng, Xiaoli Dong, Liming Zhao

**Affiliations:** Department of Neurology, Guangdong Sanjiu Brain Hospital, Guangzhou, 510510 China

**Keywords:** Medium-chain triglyceride ketogenic diet, Ketogenic diet, Drug-resistant epilepsy, Efficacy, Safety

## Abstract

**Background:**

Medium-chain triglyceride ketogenic diet (MCTKD) is  previously less commonly used in China. This study was aimed to assess the efficacy and safety of the modified MCTKD in the treatment of drug-resistant epilepsy in Chinese patients.

**Methods:**

Patients with drug-resistant epilepsy were enrolled to receive treatment with modified MCTKD in Guangdong Sanjiu Brain Hospital during December 2020 and September 2022. The modified MCTKD contained fat that provided 50–70% of the total energy, as well as proteins and carbohydrates that provided 20–30% and 20% of energy, respectively. The fat component was composed of 20–30% medium-chain triglycerides (MCTs) and 30–40% long-chain triglycerides. The efficacy and safety of the diet were assessed at 1, 3 and 6 months.

**Results:**

A total of 123 patients aged 2.5 to 65 years, were included in this study. The response rates at 1, 3 and 6 months were 49.6%, 43.1%, and 30.9%, respectively. The seizure freedom rates at 1, 3 and 6 months were 12.2%, 10.6%, and 6.5%, respectively. The retention rates at 1, 3 and 6 months were 98.4%, 65.0% and 33.3% respectively. Side effects occurred in 21.14% of patients, which were predominantly gastrointestinal symptoms such as abdominal pain, diarrhea, vomiting, and constipation, and most of them resolved after dietary adjustments. A total of 82 patients (66.7%) discontinued the treatment with the reason of refusing to eat (8.1%), poor efficacy (35.0%), poor compliance (4.9%), and inability to follow-up (9.8%). Only 4 patients (3.3%) withdrew the diet due to side effects.

**Conclusions:**

The modified MCTKD with MCTs providing 20–30% of energy has a good safety in patients with drug-resistant epilepsy, but its effectiveness needs to be enhanced. Further modifications of MCTKD with an optimal energy ratio are required to achieve a better efficacy and safety.

## Background

Ketogenic diet (KD) is a special formula diet with high fat, low carbohydrates, appropriate proteins and other nutrients. It was first proposed by Wilder in 1921 for the treatment of epilepsy [1], and was introduced to China in 2004. It is currently recommended as a safe and effective dietary treatment for drug-resistant epilepsy [2]. Over the past 100 years, various dietary protocols of KD have been proposed, which can be divided into four categories: classic ketogenic diet (CKD), medium-chain triglyceride ketogenic diet (MCTKD), modified Atkins diet, and low glycaemic index treatment [3].

The CKD has strict limitations on carbohydrate intake, limiting its implementation. MCTKD was first introduced by Huttenlocher in 1971 [4]. In MCTKD, the medium-chain triglycerides (MCTs) serve as the predominant source for ketone production. Notably, this approach does not require caloric restriction and allows a more higher carbohydrate intake of up to 20%. It has been shown that MCTKD has similar efficacy with CKD [5, 6]. In classic MCTKD, the MCTs account for 60% of the overall energy provision, and are frequently associated with gastrointestinal side effects including diarrhea, vomiting, bloating, and cramps [7–9]. For this reason, a modified MCTKD was developed, in which the MCTs provided 30% of energy and long-chain triglycerides (LCTs) provided an additional 30% of energy. This protocol achieved good results [10]. However, currently there is only a small number of studies on modified MCTKD, and the number is even smaller in China. In clinical practice, we found that MCTKD is poorly tolerated when the MCTs provide more than 40% of energy as they would cause adverse reactions. Therefore, in this study, we aimed to assess the efficacy and safety of a modified MCTKD comprising MCTs providing 20–30% of the total energy in Chinese patients with drug-resistant epilepsy.

## Methods

### Study design and patients

Patients with drug-resistant epilepsy were enrolled from Guangdong Sanjiu Brain Hospital, and received treatment with the modified MCTKD between December 2020 and September 2022. The study was approved by the Medical Ethics Committee of Guangdong Sanjiu Brain Hospital, and informed consent was obtained from the patients or their guardians (protocol number: 2022-010-003 and 2020-010-07).

### Participant inclusion/exclusion criteria

Inclusion criteria: (1) diagnosis of drug-resistant epilepsy as defined by the International League Against Epilepsy in 2010: failure of adequate trials of two tolerated appropriately chosen and used anti-seizure medications (ASMs) schedules (whether as mono therapies or in combination) to achieve sustained seizure freedom; (2) age over 2 years;  and (3) a minimum average seizure frequency of once every three months prior to enrollment.

Exclusion criteria: (1) patients with fat metabolism-related liver diseases or other metabolic disorders; (2) active fever or infectious diseases; (3) contraindications for KD treatment: diseases that interfere with glucose and fat metabolism or hepatic metabolic diseases (such as carnitine deficiency, mitochondrial enzyme deficiency, ketotic hypoglycemia, etc.); (4) patients with significant digestive, cardiovascular, respiratory, urinary system diseases or immune deficiency.

Withdrawal criteria: (1) intolerable adverse reactions; (2) lost to follow-up; (3) patient’s or parental decision to discontinue the diet for various reasons, including dissatisfaction with treatment outcomes, adverse reactions, refusal to eat, economic constraints, etc.

### KD protocol

In this study, the modified MCTKD had a fat-to-carbohydrate plus protein ratio of 1:1–2:1. Fat comprised 50–70% of total energy intake, including 20–30% for MCTs and 30–40% for LCTs, with protein contributing to 20–30% and carbohydrates providing the remaining 20%. Water intake was not restricted. MCT powder was taken with meals, and the intake amount progressively increased to reach the target contribution of 20–30% of total energy intake within a month. The intake of LCTs, proteins, and carbohydrates came from foods like meat, eggs and grains, etc. Urine ketones were monitored daily to sustain the urine ketones level at (+ +) or higher. During the treatment, daily multivitamins and minerals were supplemented according to the recommended dietary allowance for Chinese residents. For instance, a 6-year-old girl weighing 24 kg with a daily caloric requirement of 1400 kcal would receive 420 kcal (30%) from the MCT powder, divided into three servings of 15 g per meal.

### Observation indicators

The demographic and clinical profiles, etiology, type and frequency of seizures, age of epilepsy onset, age of KD initiation, duration of epilepsy before KD initiation, the usage of ASMs and examination results were recorded.

The safety indicators included full blood examination, liver and renal function tests, electrolytes, fasting glucose and fasting lipid profiles, were recorded at the 1st month of KD treatment. At the 3 and the 6 months, in addition to the aforementioned tests, data from tests of mineral levels, renal tract ultrasound, bone density testing and electroencephalography were also recorded. Side effects and corresponding measures were documented.

According to the changes in seizure frequency after KD treatment, the efficacy was categorized as: (1) seizure freedom: 100% reduction; (2) responder: equal to or more than 50% reduction; (3) ineffective: less than 50% reduction or no change in seizure frequency.

### Data analysis

Data were analyzed using SPSS 22.0 software. Measurement data are presented as median (25th percentile, 75th percentile). Categorical variables are expressed by frequency and percentages. Chi-square test was used for inferential analyses of categorical variables. Significance was set at 0.05 (two-tailed).

## Results

### Baseline characteristics

**Fig. 1:**
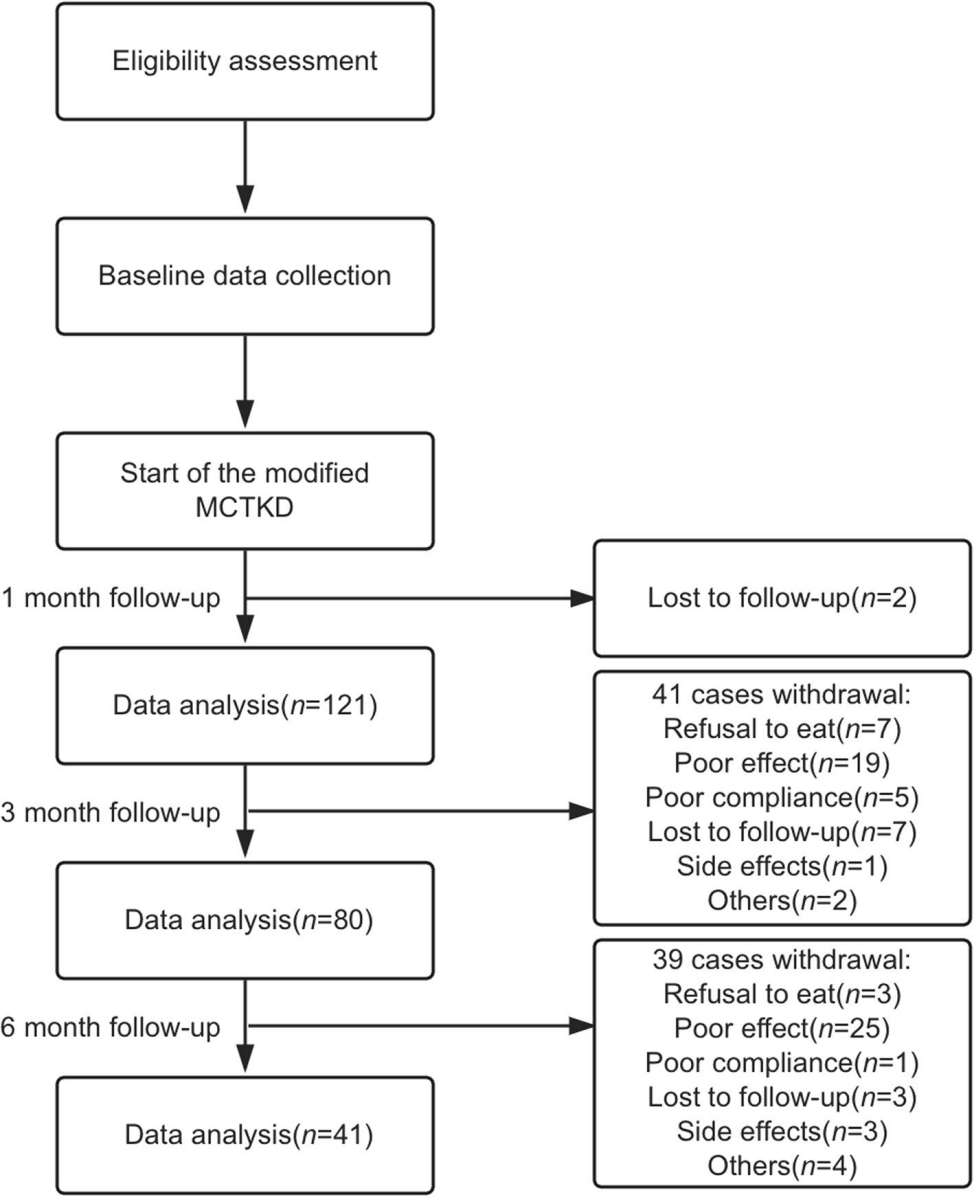
Flowchart of the study

 A total of 123 patients (73 males and 50 females, range 2.5 – 65 years) were enrolled, comprising 38 patients aged under 10 years, 31 aged 10 – 18 years, and 54 adults > 18 years. The age of epilepsy onset ranged from 3 months to 62 years. The seizure types included epileptic spasms, tonic seizure, generalized tonic-clonic seizures (GTCS), absence seizures, myoclonic seizures and focal seizures. Focal epilepsy was diagnosed in 69.9% (86 cases) of the patients, while 4.9% (6 cases) had generalized epilepsy. Additionally, there were 9 cases (7.3%) of infantile epileptic spasms syndrome, 3 cases of Lennox-Gastaut syndrome, 2 cases of Dravet syndrome, 1 case of hypothalamic hamartoma, and 1 case of Rasmussen syndrome. The demographics and clinical characteristics of the participants are detailed in Table 1. Eighty-two patients were lost or withdrawal from the study during the 6 months of follow up. The flowchart of the study is shown in Figure 1.

**Table 1:** Demographics and clinical characteristics of the 123 patients recruited

	Median/ Number	25th percentile,75th percentile/ Percentage
Age (years)	14.8	9.0, 25.9
Male sex	73	59.3%
Age of epilepsy onset (years)	7.0	2.0, 15.0
Disease duration (years)	6.4	2.5, 10.9
Monthly seizure frequency		
1–10	72	58.5%
11–50	12	9.8%
51–100	22	17.9%
> 100	17	13.8%
Epilepsy type		
Focal epilepsy	86	69.9%
Generalized epilepsy	6	4.9%
Unknown	31	25.2%
Syndrome		
IESS	9	7.3%
LGS	3	2.4%
Dravet syndrome	2	1.6%
Hypothalamic hamartoma	1	0.8%
Rasmussen syndrome	1	0.8%
Medication usage		
2	51	41.5%
3	40	32.5%
4	25	20.3%
5 or more	7	5.7%

### Effectiveness and retention rates

At 1, 3 and 6 months after the start of the modified MCTKD, 121, 80 and 41 patients remained on the diet, with a retention rate of 98.4%, 65.0% and 33.3%, respectively. At these time points, the responder rates were 49.6% (61 cases), 43.1% (53 cases) and 30.9% (38 cases), respectively; while 12.2% (15 cases), 10.6% (13 cases) and 6.5% (8 cases) achieved seizure freedom, respectively. There were no significant differences in the responder rates, seizure freedom rate and retention rate across different age groups (Table 2).

**Table 2:** Effectiveness of the modified MCTKD and the retention rates at 1, 3 and 6 months of follow-up

		1 month			3 months			6 months		
Groups	cases	Responder rate (%)	Seizure freedom(%)	Retention rate(%)	Responder rate(%)	Seizure freedom(%)	Retention rate(%)	Responder rate(%)	Seizure freedom(%)	Retention rate(%)
< 10 years	38	17 (44.7%)	3 (7.9%)	37 (97.4%)	15 (39.5%)	3 (7.9%)	21 (55.3%)	14 (36.8%)	2 (5.3%)	14 (36.8%)
10-18years	31	12 (38.7%)	3 (9.7%)	30 (96.8%)	13 (41.9%)	3 (9.7%)	22 (71.0%)	11 (35.5%)	2 (6.5%)	12 (38.7%)
≥ 18 years	54	32 (59.3%)	9 (16.7%)	54 (100.0%)	25 (46.3%)	7 (13.0%)	37 (68.5%)	13 (24.1%)	4 (7.4%)	15 (27.8%)
total	123	61 (49.6%)	15 (12.2%)	121 (98.4%)	53 (43.1%)	13 (10.6%)	80 (65.0%)	38 (30.9%)	8 (6.5%)	41 (33.3%)
*P* value		0.144	0.465	0.313	0.823	0.756	0.315	0.354	1.000	0.523

### Safety

Among the 123 patients, a total of 26 cases (21.14%) reported side effects, including diarrhea (4.07%), abdominal pain (3.25%), simultaneous occurrences of diarrhea and abdominal pain (2.44%), vomiting (1.63%), constipation (3.25%), fever (0.81%), rash (0.81%), hyperuricemia (1.63%), allergy (0.81%), iron deficiency (0.81%) and fatigue (1.63%). Gastrointestinal symptoms were the most frequently reported adverse events (Table 3). There was a significant difference in incidence of side effects between the <18 years group (14.49%) and the > 18 years group (29.63%) (χ^2^ = 4.164, *P* = 0.041).

**Table 3:** Side effects of the modified MCTKD in patients of different age groups

Side effects	Cases (incidence)			
	< 18 years (69 cases)	≥ 18 years (54 cases)	Total (123 cases)	*P value*
Diarrhea	1 (1.45%)	4 (7.41%)	5 (4.07%)	
Abdominal pain	2 (2.90%)	2 (3.70%)	4 (3.25%)	
Diarrhea&abdominal pain	0 (0.00%)	3 (5.56%)	3 (2.44%)	
Vomiting	1 (1.45%)	1 (1.85%)	2 (1.63%)	
Constipation	2 (2.90%)	2 (3.70%)	4 (3.25%)	
Fever	0 (0.00%)	1 (1.85%)	1 (0.81%)	
Rash	0 (0.00%)	1 (1.85%)	1 (0.81%)	
Hyperuricemia	2 (2.90%)	0 (0.00%)	2 (1.63%)	
Allergy	0 (0.00%)	1 (1.85%)	1 (0.81%)	
Iron deficiency	1 (1.45%)	0 (0.00%)	1 (0.81%)	
Fatigue	1 (1.45%)	1 (1.85%)	2 (1.63%)	
Total	10 (14.49%)	16 (29.63%)	26 (21.14%)	0.041

Regarding lipid profiles, 89 patients underwent evaluations before and after 3-month treatment. In 61 cases (68.5%), lipid levels remained within normal ranges both pre- and post-treatment. Nineteen cases (21.3%) showed modest increases in total cholesterol (TC), triglyceride (TG), low density lipoprotein cholesterol (LDL) or a combination of them. Nine patients (10.1%) with initially elevated lipid levels showed normalization at the 3-month follow-up. Additionally, one patient showed increased TC but reduced LDL following the treatment.

### Reasons for withdrawal

A total of 82 patients (66.7%) discontinued the treatment due to the reasons of refusing to eat (10 cases, 8.1%), poor efficacy (44 cases, 35.8%), poor compliance (6 cases, 4.9%), and inability to follow-up (12 cases, 9.8%). The side effects led to KD withdrawal in 4 patients (3.3%), including two cases of respiratory tract infections, one case of menstrual irregularities, and one case of hair loss (Table 4).

**Table 4:** Reasons for the withdrawal from the modified MCTKD during the 6 months of follow-up

Reasons for withdrawal	Within 1 month	1-3 months	3–6 months	Total
Refusal to eat	0	7	3	10
Surgery	0	1	1	2
Cerebellar atrophy	0	1	0	1
Effective seizure control	0	0	2	2
Respiratory tract infection	0	1	1	2
Preparation for pregnancy	0	0	1	1
Menstrual irregularities	0	0	1	1
Hair loss	0	0	1	1
Poor effect	0	19	25	44
Poor compliance	0	5	1	6
Loss of follow-up	2	7	3	12
Total	2	41	39	82

## Discussion

The CKD predominantly derives it energy between 60–80% of energy from long-chain fatty acids (LCFAs), which have a chain length of 16 to 20 carbon atoms. This regiment sets strict restrictions on the content of carbohydrates and the daily energy intake. In contrast, the MCTKD mainly provides energy through medium-chain fatty acids (MCFAs) with carbon chain lengths ranging from 6 to 12 carbon atoms. Dietary MCTs are broken down in the gastrointestinal tract by lipases into MCFAs, including 6-carbon caproic acid (1–2%), 8-carbon octanoic acid (65–75%), 10-carbon decanoic acid (25–35%) and 12-carbon lauric acid (1–2%), which are then absorbed directly through the gut wall and transferred to the liver, where the MCFAs undergo β-oxidation to produce β-hydroxybutyrate, acetoacetate and acetone. LCFAs are transported in the form of chylomicrons, entering the bloodstream through the lymphatic circulation and distributed throughout the body. In contrast, MCFAs are directly shuttled to the liver via the portal venous system, bypassing both chylomicron formation and extensive peripheral distribution. Moreover, MCFAs are rarely involved in de novo fatty acid synthesis and less likely to accumulate in the body. MCFAs possess a more efficient digestive and hydrolytic profile compared to LCFAs. They have strong emulsification capacity and can promote the emulsification of LCFAs [11].

In classic MCTKD, MCTs provide 60% of the total energy, which might cause abdominal symptoms, restricting its applications. For a better tolerance, in the modified MCTKD, MCTs provide 30% of energy while LCTs provide 30% of energy [5, 10]. Our study was the first study in China to assess the efficacy and safety of modified MCTKD in which the MCTs providing 20–30% of energy. This MCTKD study also had the largest sample size to date. The patients included in the study had diverse demographics, including age, types of epilepsy, and disease duration. At the 1, 3, and 6 months of follow-up, the responder rates were 49.6%, 43.1%, and 30.9%, respectively, which were lower than those in most classic MCTKD studies [4, 7, 8] (Table 5). In 1989, Schwartz et al. reported a pioneer study in which a modified MCTKD was administered to 12 patients, achieving a 66.7% responder rate, comparable to that of the classic MCTKD [10]. In Liu’s study, the children were divided into three groups in which they received 40–55% MCTs, 60% MCTs, and >60% MCTs diet. Results suggested that 21% of children were seizure free, 19% had a >90% seizure reduction, and another 42% had a 50–90% seizure reduction [12]. Neal and colleagues conducted the first randomized study comparing efficacy of CKD with that of MCTKD in which MCTs provided 40–50% of energy. Their findings revealed responder rates of 29.6% at 3 months and 19.4% at 6 months in the MCTKD group, which were lower than those in our study. Additionally, no significant differences were observed between the two groups in achieving over 50% or 90% seizure reduction [5]. In a Thai study, MCTs supplying 50% of energy showed a  similar responder rate in children to ours at 1 month, but contrary to our results, their effectiveness improved at 3 months [13]. In the study conducted by Lowe et al, MCTs constituting 40–50% of energy resulted in a responder rate of 58.8%, which was slightly higher than ours [6]. Also, the effectiveness rates in our study were lower than that of the CKD studies in children and adult patients [14–16]. Several factors may account for the lower efficacy in our study. First, our study used urine ketone testing as the monitoring indicator due to its ease of use and cost-effectiveness; however, it may not correlate as closely as serum ketones with seizure control [17, 18]. Second, it has been found that decanoic acid and octanoic acid play important roles in the antiseizure effects of MCTKD, which might limit the utility of ketone monitoring. These two factors could contribute to suboptimal monitoring potentially resulting in lower efficacy rates. Third, there is considerable heterogeneity amongst patient populations across different studies. Last, the recruitment for this study was during the COVID-19 pandemic, which might lead to decreased patient adherence. Therefore, future large-scale studies are needed to investigate the use ketone bodies to monitor the efficacy of MCTKD and to identify more effective biomarkers for surveillance. Additionally, our analysis revealed no statistically significant disparity in the effectiveness rate across different age groups, suggesting that modified MCTKD is applicative for both pediatric and adult patients.

**Table 5:** Previous MCTKD clinical studies

Study	*n*	Age (years)	MCT energy ratio	Duration (months)	Responder rate	Seizure freedom rate	Side effects
Huttenlocher et al., 1971 [4]	12	2.5–16	60%	2.5–13	66.7%	50%	41.7%
Trauner, 1985 [7]	17	1–13	60%	2–60	58.8%	29.4%	29.4%
Sills et al., 1986 [8]	50	2–15	45–60%	6–48	50.0%	18.2%	-
Schwartz et al., 1989 [10]	12 27	5–54	30% 60%	1.5–48	66.7% 81.7%	-	~ half
Liu, 2008 [12]	43	2–16	40–55% 60% > 60%	-	82%	21%	-
Neal et al., 2009 [5]	72	2–16	40–45%	12	29.2%, 19.4%, and 22.2% at 3, 6, and 12 months	-	-
Chomtho et al., 2016 [13]	16	0.5–16	50%	3	50%, and 64.3% at 1 and 3 months	6.25%, and 28.6% at 1 and 3 months	80%
Lowe et al., 2022 [6]	17	4.0–8.5	40–50%	6	58.8%	-	82.4%

The wide occurrence of side effects has historically hindered the full application of the MCTKD. However, in our study, the incidence of side effects was notably lower than that reported in most other MCTKD studies [4, 6, 7, 10, 13], and even surpassed the tolerability profiles of CKD [19–21]. Consistent with other MCTKD studies, gastrointestinal symptoms were the predominant side effects observed, such as abdominal pain and diarrhea. After dietary adjustments, most gastrointestinal symptoms resolved, and no serious adverse reactions were documented in our study. Our data also showed that adults experienced side effects more frequently than children and adolescents, suggesting that children and adolescents patients have a higher tolerance for the modified MCTKD.

The retention rate was modest [5, 6], and showed a marked decline over time in this study. Retention is influenced by multiple factors, including the efficacy of the diet, the side effects, confidence in the therapy, economic constraints, and ingrained dietary preferences. The decreased retention rates might be caused by suboptimal treatment outcomes, even though the low rate of adverse effects. In future studies, we aim to identify an optimal ratio of MCTs that could achieve the best effectiveness while maintaining the safety.

Considering that KD is a high-fat diet, the lipid profiles of patients were analyzed in detail. Results showed that 68.5% of patients maintained normal lipid levels both pre- and post-intervention, indicating that the modified MCTKD exerts minimal impact on blood lipids in most patients. Intriguingly, while 21.3% of patients exhibited elevated blood lipids following treatment, 10.1% of patients actually presented with reduced lipids, highlighting the potential of dual effects of the modified MCTKD on lipid profiles. The capacity of modified MCTKD to lower blood lipid levels in this study was consistent with findings from previous KD research [22, 23]. A patient in this study displayed increased TC, decreased LDL and elevated high-density lipoprotein (HDL) after treatment, which coincided with the study by Liu YM, et al [24], in which the MCTKD had a positive effect on lipid profiles compared to CKD in children with intractable epilepsy, lowering TC/HDL ratios.

In MCTKD, it has been established that decanoic acid and octanoic acid have antiseizure effects. Studies in animal models have shown that decanoic acid inhibits the epileptiform activity. After intake of MCTKD, decanoic acid rapidly crosses the blood–brain barrier to reach the brain at sufficient concentrations to reduce neuronal excitation, thereby reducing seizure frequency in animals [25]. Decanoic acid exerts its antiseizure effects through multiple pathways. One such mechanism involves the direct and selective inhibition of AMPA receptors in animal models, a process which has shown promise in the treatment of focal seizures and GTCS. The site where decanoic acid acts on AMPA receptors is different from that of the anti-seizure medication perampanel, suggesting a novel pathway of intervention [26–28]. Additionally, decanoic acid is found to activate the peroxisome proliferator-activated receptor gamma (PPARγ), promoting mitochondrial function by stimulating mitochondrial biogenesis and increasing the mitochondrial complex I activity [29–32]. Octanoic acid, which is the most abundant MCFA after dietary MCTs broken down, has been observed to elevate the threshold for myoclonic and clonic convulsions in rats [33]. Octanoic acid has different antiseizure mechanisms from decanoic acid. It does not inhibit PPARγ in vitro or augment mitochondrial function in vivo [29, 30, 34]. Unlike decanoic acid, octanoic acid cannot inhibit AMPA receptors. The mechanisms of its antiseizure effect are hypothesized to be through indirect pathways, potentially involving modulatory effects on adenosine receptors [26].

## Conclusions

The modified MCTKD with MCTs providing 20– 30% of energy has a good safety in patients with drug-resistant epilepsy, but the effectiveness needs to be enhanced. Further studies are needed to modify the energy ratio of MCTKD to achieve betterr efficacy and lower adverse effects.

## Data Availability

The datasets used and analyzed during the current study are available from the corresponding author on reasonable request.

## Abbreviations

ASMs: Anti-seizure medications
CKD: Classic ketogenic diet
KD: Ketogenic diet
LCFAs: Long-chain fatty acids
LCTs: Long-chain triglycerides
MCFAs: Medium-chain fatty acids
MCTs: Medium-chain triglycerides
MCTKD: Medium-chain triglyceride ketogenic diet
